# PINK1/Parkin-mediated mitophagy inhibits warangalone-induced mitochondrial apoptosis in breast cancer cells

**DOI:** 10.18632/aging.202965

**Published:** 2021-04-30

**Authors:** Lianzhi Mao, Huahuan Liu, Rongjun Zhang, Yudi Deng, Yuting Hao, Wenzhen Liao, Miaomiao Yuan, Suxia Sun

**Affiliations:** 1 Department of Nutrition and Food Hygiene, Guangdong Provincial Key Laboratory of Tropical Disease Research, School of Public Health, Southern Medical University, Guangzhou 510515, Guangdong, China; 2 Cancer Research Institute, School of Basic Medical Sciences, Southern Medical University, Guangzhou 510515, Guangdong, China; 3 The Eighth Affiliated Hospital, Sun Yat-Sen University, Shenzhen 518033, Guangdong, China

**Keywords:** warangalone, apoptosis, mitophagy, PINK1/Parkin, breast cancer

## Abstract

Breast cancer is the most common malignancy in women all around the world, especially in many countries in Asia. However, antitumor drugs with unique curative effects and low toxic side-effects have not been found yet. Warangalone is an isoflavone extracted from the *Cudrania tricuspidata* fruit, and is reported to possess anti-inflammatory and anti-cancer activity. The purpose of this study was to determine the effects of warangalone on breast cancer cells. In this study, we found that warangalone decreased the viability of breast cancer cells by increasing the generation of reactive oxygen species (ROS) resulting in mitochondrial damage and decreased mitochondrial membrane potential (MMP). Warangalone induced mitochondrial apoptosis by increasing the BAX/BCL-2 ratio. Warangalone activated mitophagy via upregulation of PINK1 and Parkin expression and co-localization. The combination of warangalone and autophagy inhibitors or PINK1 siRNA increased the degree of cell apoptosis compared to treatment with warangalone alone. Warangalone damages mitochondria via ROS, thereby triggering PINK1/Parkin-mediated mitophagy and inducing mitochondrial apoptosis. However, autophagy/mitophagy protects against warangalone-induced mitochondrial apoptosis. A combination of warangalone and autophagy/mitophagy inhibitors may be a potential treatment for breast cancer.

## INTRODUCTION

Breast cancer is the most common malignancy and the second leading cause of cancer-related death in women [1]. Breast cancer is an important life-threatening malignancy for women in most Asian countries [2], and its incidence is increasing more rapidly in Asian countries than in Western countries [2, 3]. Based on cell surface hormone receptors, breast cancer can be divided into 4 subtypes: Luminal A-estrogen receptor positive (ER^+^), Luminal B-progesterone receptor positive (PR^+^), human epidermal growth factor receptor 2 positive (HER2^+^), and triple-negative breast cancer (TNBC). TNBC, which lacks expression of all 3 hormone receptors is an aggressive malignancy with poor prognosis, and accounts for 10-20% of breast cancer cases [4].

Breast cancer is treated with mastectomy, chemotherapy, and radiation therapy, and all have profound side-effects [5]. Compared with mastectomy and radiation therapy, chemotherapy is more effective for metastatic cancer [6]. However, traditional chemotherapy drugs are associated with serious side-effects such as neutropenia, stomatitis and mucositis [7]. Nowadays, nanoparticle-based vaccine delivery for cancer is developing, but still in its infancy [8]. Thus, the development of natural antitumor drugs with unique curative effects and low toxic side-effects has become an important area of research, such as the synthesis of 14’,15’-dehydro-ritterazine Y 5 and solasodine acetate 2 [9, 10].

*Cudrania tricuspidata* (*Carr*.) is a deciduous mulberry tree that primarily grows in China [11]. Studies have reported that bioactive components extracted from *Carr*. exhibit hepatoprotective [12], anti-oxidant [13] and anti-cancer effects [14]. However, there has been little study of the bioactive components of the fruit of *Cudrania tricuspidata*. The fruit is an edible food and used as a brewing ingredient, but is also used as a traditional medicine for the treatment of trauma and bruises. Warangalone, also referred to as “scandenolone”, is a type of isoflavone that is extracted from the fruit. Recent studies have reported that warangalone possesses anti-inflammatory [15], anti-diabetes [16] and anti-cancer properties [17, 18]. Hu et al. [17] reported that warangalone inhibited cell proliferation and invasion of human melanoma cells through inducing apoptosis and blocking autophagy flux. Jiang et al. [18] reported that warangalone decreased the viability of ER-positive MCF7 cells. However, few studies have examined warangalone as a potential treatment for breast cancer.

Inducing tumor cell apoptosis is the primary method by which chemotherapy drugs kill tumor cells, and mitochondria plays a crucial role in the process of apoptosis [19]. In the early stage of apoptosis, the BCL-2 family protein BAX is translocated to mitochondria, where it opens the mitochondrial surface osmotic conversion pores; this subsequently causes the mitochondrial transmembrane potential to collapse. Then, cytosolic solutes enter the mitochondrial body due to a high osmotic pressure, causing the mitochondrial matrix to swell and the outer membrane of the mitochondria to rupture which releases the apoptosis factor cytochrome C into the cytoplasm, which in turn triggers cell death [19, 20].

On the other hand, tumor cells also undergo autophagy which may protect tumor cells from apoptosis. Mounting evidence suggests that after anti-tumor drugs act on tumor cells, the stress induces autophagy which reduces the efficacy of the anti-tumor drugs [21]. With respect to mitochondria, mitophagy is a type of autophagy that selectively clears damaged mitochondria, and thereby affects cell apoptosis. There are two signaling pathways for mitophagy; ubiquitin-dependent and ubiquitin-independent [22]. Ubiquitin-dependent mitophagy is regulated by the phosphatase and tensin homologue (PTEN)-induced putative kinase 1 (PINK1)/Parkin pathway, which plays an important role in the elimination of dysfunctional organelles via various mitochondrial physiological properties [22]. It has recently been reported that multidrug-resistant cancer cells become more sensitive to chemotherapy drugs when PINK1/Parkin-dependent mitophagy is inhibited [23]. As such, inducing apoptosis combined with inhibiting mitophagy may be a potential strategy for the treatment of breast cancer. The purpose of this study was to determine the effects of warangalone on breast cancer cells from the perspective of apoptosis and mitophagy.

## RESULTS

### Warangalone inhibits the viability of breast cancer cells

**Figure 1:**
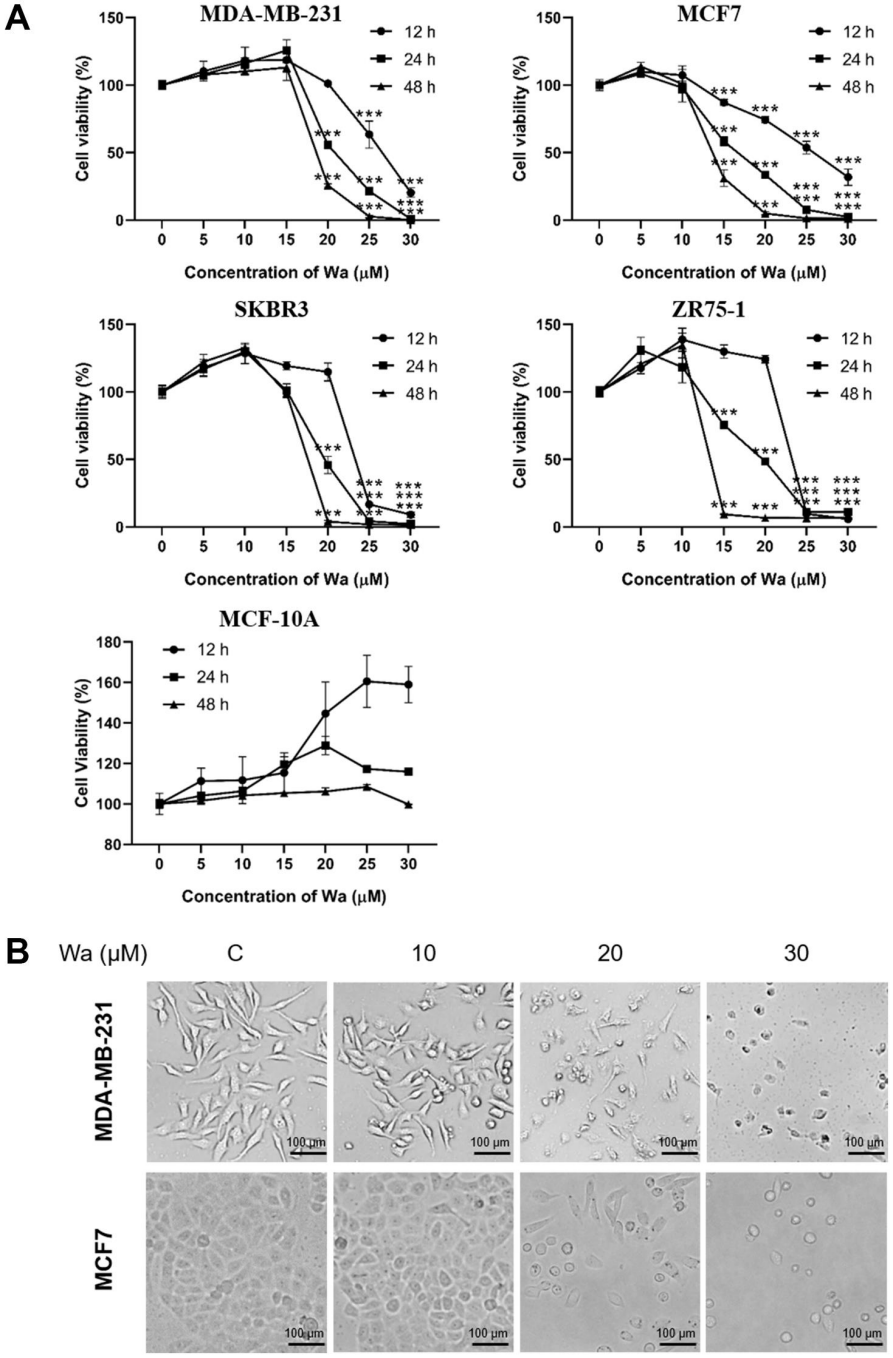
**Warangalone decreases the viability of breast cancer cells.** (**A**) MDA-MB-231, MCF7, SKBR3, ZR75-1 and MCF-10A cells were treated with warangalone of different concentrations for 12, 24, and 48 h. Cell viability was detected by MTT assay. One-way ANOVA was used to compare between the control and warangalone treatment groups (n ≥ 3). *P < 0.05, **P < 0.01, ***P < 0.001 compared to the respective control group. (**B**) MDA-MB-231/MCF7 cells were treated with 0, 10, 20 and 30 μM warangalone for 24 h. Cell morphology was observed by microscopy.

To study the cytotoxic effects of warangalone, 4 breast cancer cell lines and 1 normal breast cell line were exposed to warangalone for 12, 24 and 48 h. Cell viability was detected with the MTT assay. Warangalone significantly reduced MTT tetrazolium salt formation in all 4 cell lines (MDA-MB-231, MCF-7, ZR75-1, and SKBR3) in a concentration- and time-dependent manner (Figure 1A). In MDA-MB-231 cells, the lowest concentration of warangalone that significantly inhibited proliferation was 20 μM, while in MCF-7 cells the lowest warangalone concentration that inhibited proliferation was 15 μM. However, instead of inhibiting proliferation, warangalone promoted proliferation in normal breast cell lines (MCF-10A) (Figure 1A).

Cellular morphology after treatment with warangalone (0, 10, 20 and 30 μM) for 24 h was observed. As shown in Figure 1B, after treatment with 20 μM or 30 μM warangalone, the numbers of MDA-MB-231 and MCF7 cells were obviously decreased, which is consistent with the results of the MTT assay. The cells appeared shrunken and round after treatment with warangalone. This appearance is similar to that during apoptosis, suggesting that warangalone might induce apoptosis.

### Warangalone leads to mitochondrial damage via ROS

**Figure 2:**
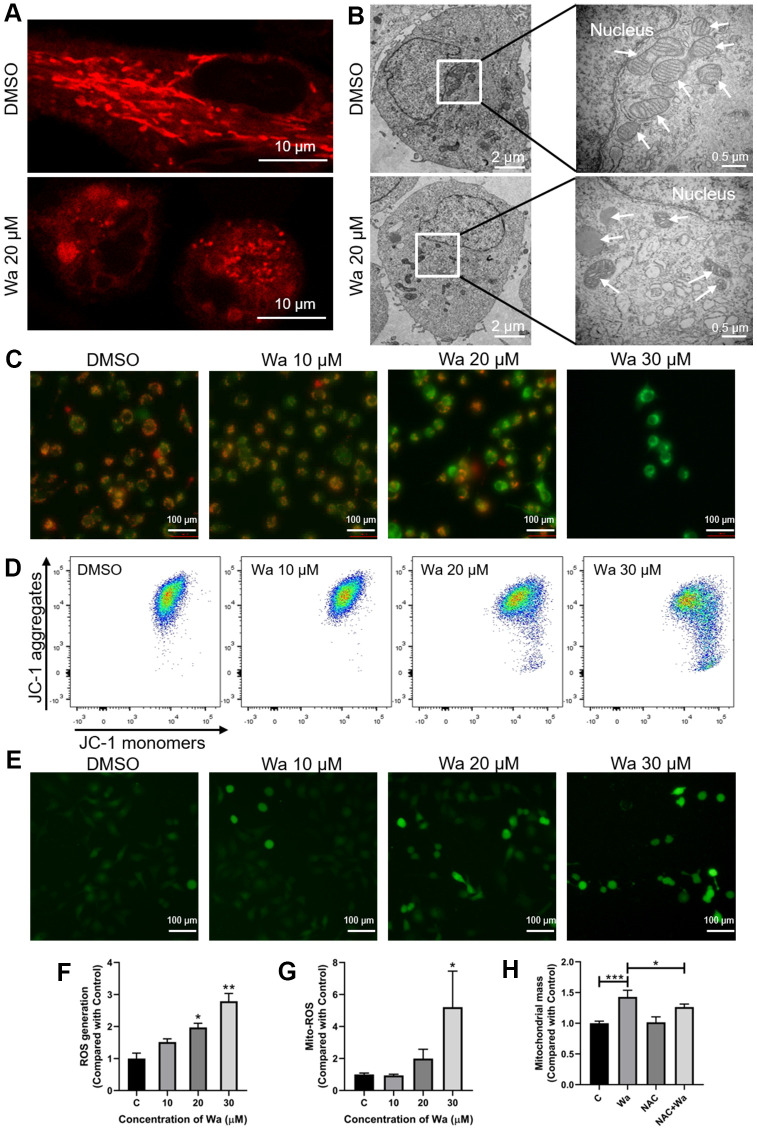
**Warangalone causes to mitochondrial damage via ROS.** (**A**, **B**) MDA-MB-231 cells were treated with 20 μM warangalone for 12 h. The mitochondria were stained with MitoTracker™ Red CMXRos 30 min, and observed by (**A**) confocal laser scanning microscopy (CLSM) (mitochondria emit red) and (**B**) transmission electron microscopy (TEM) (arrows mark mitochondria). (**C**, **D**) MDA-MB-231 cells were treated with the indicated concentrations of warangalone for 12 h. Fluorescence microscopy (**C**) and flow cytometry (**D**) were used to detect the mitochondrial membrane potential. JC-1 aggregates emit orange, and JC-1 monomers emit green. (e-g) MDA-MB-231 cells were treated with the indicated concentrations of warangalone for 12 h. Whole cell ROS was detected through fluorescence microscope (**E**) and fluorescence spectrophotometry (**F**). ROS labeled with DCFH-DA emit green (**E**). MitoSOX was used to specifically detect mitochondrial superoxides through flow cytometry (**G**). (**H**) MDA-MB-231 cells were pretreated with NAC (5 mM) for 1 h, and cultured with warangalone (20 μM) for 12 h. The mitochondria were stained with MitoTrackerTM Red CMXRos for 30 min and flow cytometry was used to detect mitochondrial mass. One-way ANOVA was used for statistical analysis (n ≥ 3). *P < 0.05, **P < 0.01, ***P < 0.001 compared to the respective control group.

Mitochondria supply energy to maintain cellular functions, such as proliferation and migration, and normal cellular functions indicate mitochondria are functioning [19]. After treatment with warangalone for 12 h, the mitochondria in MDA-MB-231 cells became shorter and formed aggregates (Figure 2A). Compared to the control group, mitochondria in treated cells became smaller and the mitochondrial cristae decreased and even disappeared (Figure 2B). These observations indicated that warangalone treatment resulted in mitochondrial damage.

Fluorescence microscopy and flow cytometry were performed to qualitatively and quantitatively, respectively, detect MMP to confirm mitochondrial damage. Under ordinary circumstances, the MMP is high, and JC-1 forms aggregates emitting red, while under stress the MMP decreases and JC-1 aggregates are transformed into JC-1 monomers which emit green. After cells were treated with 20 or 30 μM warangalone, JC-1 dyeing with green increased and JC-1 dyeing with red decreased, which implied that JC-1 aggregates significantly changed to JC-I monomers, indicating a decrease of MMP and mitochondrial damage (Figure 2C, 2D).

Fluorescence microscopy and fluorescence spectrophotometry were used to determine if treatment with warangalone resulted in ROS generation. As shown in Figure 2E, 2F, warangalone significantly promoted the generation of ROS, suggesting that warangalone might damage mitochondria via the generation of ROS.

ROS can be generated from cells stimulated by exogenous sources such as drugs and radiation, while endogenous ROS are generated in some organelles, such as peroxisomes and mitochondria. MitoSOX, a specific mitochondrial fluorescent dye, was used to detect mitochondrial-mediated ROS generation. As shown in Figure 2G, warangalone significantly increased the generation of mitochondrial superoxide in a dose-dependent manner, indicating that the generation of ROS promoted by warangalone might be due to the release of mitochondrial superoxide. To further confirm the relations between mitochondrial damage and the generation of ROS, N-acetyl-L-cysteine (NAC) was used to inhibit ROS generation. As shown in Figure 2H, warangalone significantly increased mitochondrial mass, but NAC inhibited this change, indicating that mitochondrial damage caused by warangalone was due to ROS.

### Warangalone induces mitochondrial apoptosis

**Figure 3:**
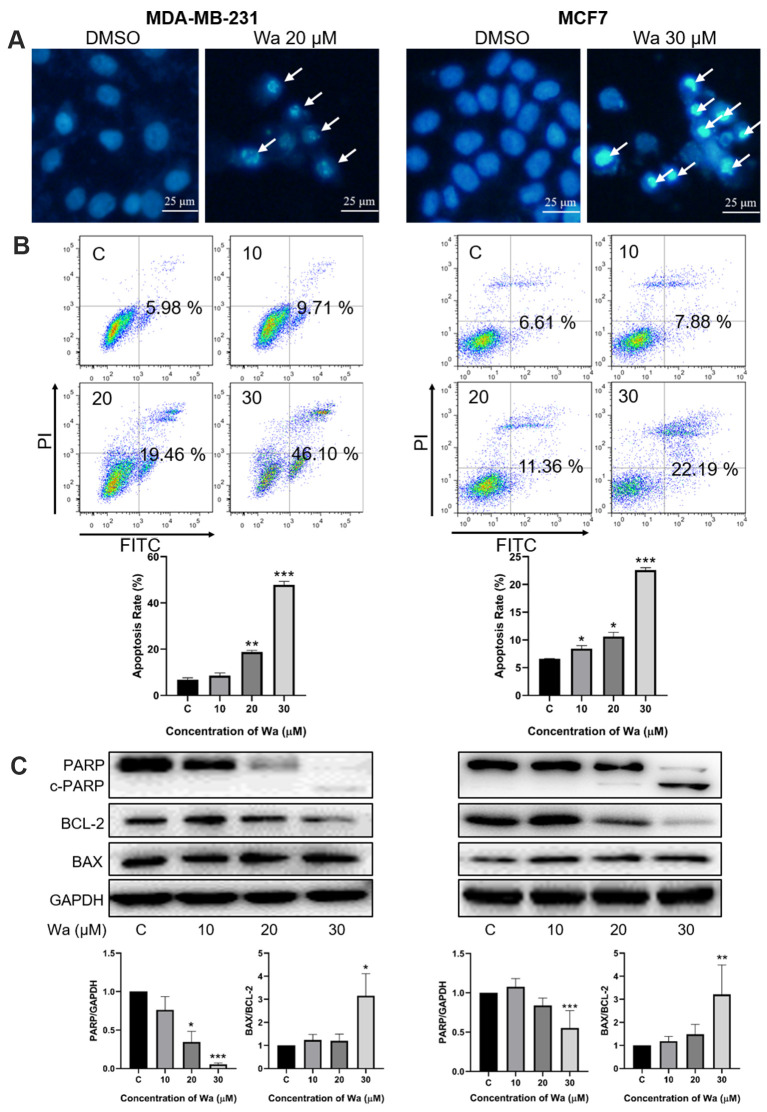
**Warangalone induces mitochondrial apoptosis.** (**A**) MDA-MB-231 and MCF7 cells were treated with 20 μM warangalone for 24 h. Treated and untreated cells were stained with Hoechst 33342 solution and the nuclei were observed with fluorescence microscopy. Arrows indicate shrunken and darker nuclei. (**B**) MDA-MB-231 and MCF7 cells were treated with the indicated concentrations of warangalone for 24 h. The apoptosis rate was detected by Annxin V-FITC/PI assay. (**C**) MDA-MB-231 and MCF7 cells were treated with the indicated concentrations of warangalone for 24 h. Representative Western blots showed the expression of PARP, BCL-2, and BAX. GAPDH was used as the loading control. Protein expression of PARP was quantified by densitometry and normalized to GAPDH (ratio PARP:GAPDH). Protein expression of BAX was quantified by densitometry and normalized to BCL-2 (ratio BAX:BCL-2). One-way ANOVA was used for statistical analysis (n ≥ 3). *P < 0.05, **P < 0.01, ***P < 0.001 compared to the respective control group.

Changes of cellular morphology suggested that warangalone induced apoptosis (Figure 1B). Thus, the Hoechst 33342 staining assay was used to determine if warangalone induced apoptosis. As shown in Figure 3A, after cells were treated with warangalone for 24 h, there were many shrunken and darker nuclei in MDA-MB-231 and MCF7 cells compared to untreated cells, indicating that apoptosis occurred.

The Annexin V/PI assay was used to examine the apoptosis rate. The apoptosis rate in MDA-MB-231 and MCF7 cells increased in a dose-dependent manner (Figure 3B). After treatment with 30 μM warangalone for 24 h, the apoptosis rate significantly increased by approximately 50% in MDA-MB-231 cells, and increased by 22% in MCF7 cells.

To examine the mechanism of apoptosis, Western blotting was performed to examine the expression levels of PARP, BAX, and BCL-2. As shown in Figure 3C, the expression level of PARP significantly decreased in a dose-dependent manner in MDA-MB-231 and MCF7 cells, indicating that PARP was cleaved to induce apoptosis. In addition, warangalone significantly increased the BAX/BCL-2 ratio in a dose-dependent manner, indicating that mitochondrial apoptosis was activated.

### Warangalone induces cell autophagy

**Figure 4:**
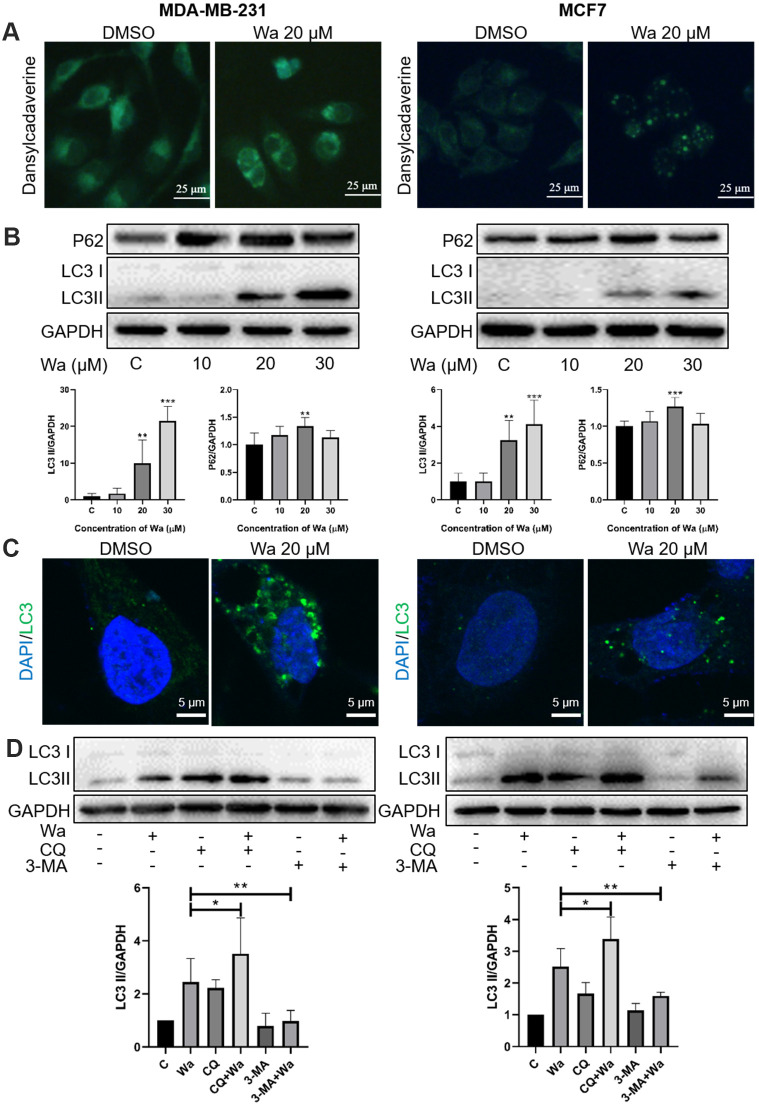
**Warangalone induces cell autophagy.** (**A**) MDA-MB-231 and MCF7 cells were treated with 20 μM warangalone for 12 h. Treated and untreated cells were stained with MDC solution and observed by fluorescence microscopy. MDC-labeled autophagic vacuoles emit bright green. (**B**) MDA-MB-231 and MCF7 cells were treated with the indicated concentrations of warangalone for 12 h. Representative Western blots show the expression of P62 and LC3. GAPDH was used as the loading control. Protein expression of P62 and LC3 II was quantified by densitometry and normalized to GAPDH (ratio of P62 or LC3 II to GAPDH). (**C**) MDA-MB-231 and MCF7 cells were treated with 20 μM warangalone for 12 h, then immunostained with LC3 antibody and stained with DAPI. Fluorescence was observed with a confocal laser scanning microscope (CLSM). LC3 labeled with Alexa Fluor 488 emits green, and nuclei labeled with DAPI emit blue. (**D**) MDA-MB-231 and MCF7 cells were pretreated with CQ and 3-MA for 1 h, and then treated with 20 μM of warangalone for 12 h. Representative Western blots show the expression of LC3. GAPDH was used as the loading control. Protein expression of LC3 II was quantified by densitometry and normalized to GAPDH (ratio LC3 II: GAPDH). One-way ANOVA was used for statistical analysis (n ≥ 3). *P < 0.05, **P < 0.01, ***P < 0.001 compared to the respective control group.

Autophagy also plays an important role in cell proliferation and cell death. To study whether warangalone induced cell autophagy, the MDC assay was first performed to observe autophagic vacuoles. As shown in Figure 4A, after cells were treated with warangalone for 12 h, there were more MDC-labeled autophagic vacuoles emitting bright green fluorescence in MDA-MB-231 and MCF7 cells as compared to the control group.

Western blotting was performed to detect the expression levels of LC3 (an autophagy marker protein) and P62 (an autophagy-associated protein). When autophagy is induced, LC3 I form first, and then conjugate to form LC3 II, which interacts with P62 in the membranes of autophagosomes. Subsequently, lysosomes fuse with autophagosomes and degrade autophagy substrate proteins, like P62. As shown in Figure 4B, warangalone significantly increased the expression level of LC3 II in a dose-dependent manner, indicating that warangalone induced cell autophagy. The expression of P62 was also upregulated by warangalone in MDA-MB-231 and MCF7 cells. However, compared with the 20 μM warangalone group, the expression of P62 in the 30 μM warangalone group was decreased, which might because of P62 degradation via autolysosomes [24]. In addition, cells treated with warangalone for 12 h exhibited more green fluorescent puncta-labeled LC3 compared to the control group, which confirmed that warangalone increased the expression of LC3 and induced cell autophagy (Figure 4C).

Chloroquine (CQ) and 3-methyladenine (3-MA) are 2 autophagy inhibitors, and were used to determine if warangalone induced autophagic flux. CQ inhibits autophagic flux by decreasing autophagosome-lysosome fusion, while 3-MA inhibits autophagy by inhibiting class III PI3K. As shown in Figure 4D, the expressions of LC3 in MDA-MB-231 and MCF7 cells in CQ + warangalone groups were higher than in the warangalone alone groups. However, the expressions of LC3 in the 3-MA + warangalone groups were lower than in the warangalone alone groups. These results indicated that warangalone induces cell autophagic flux.

### Warangalone induces cell mitophagy

**Figure 5:**
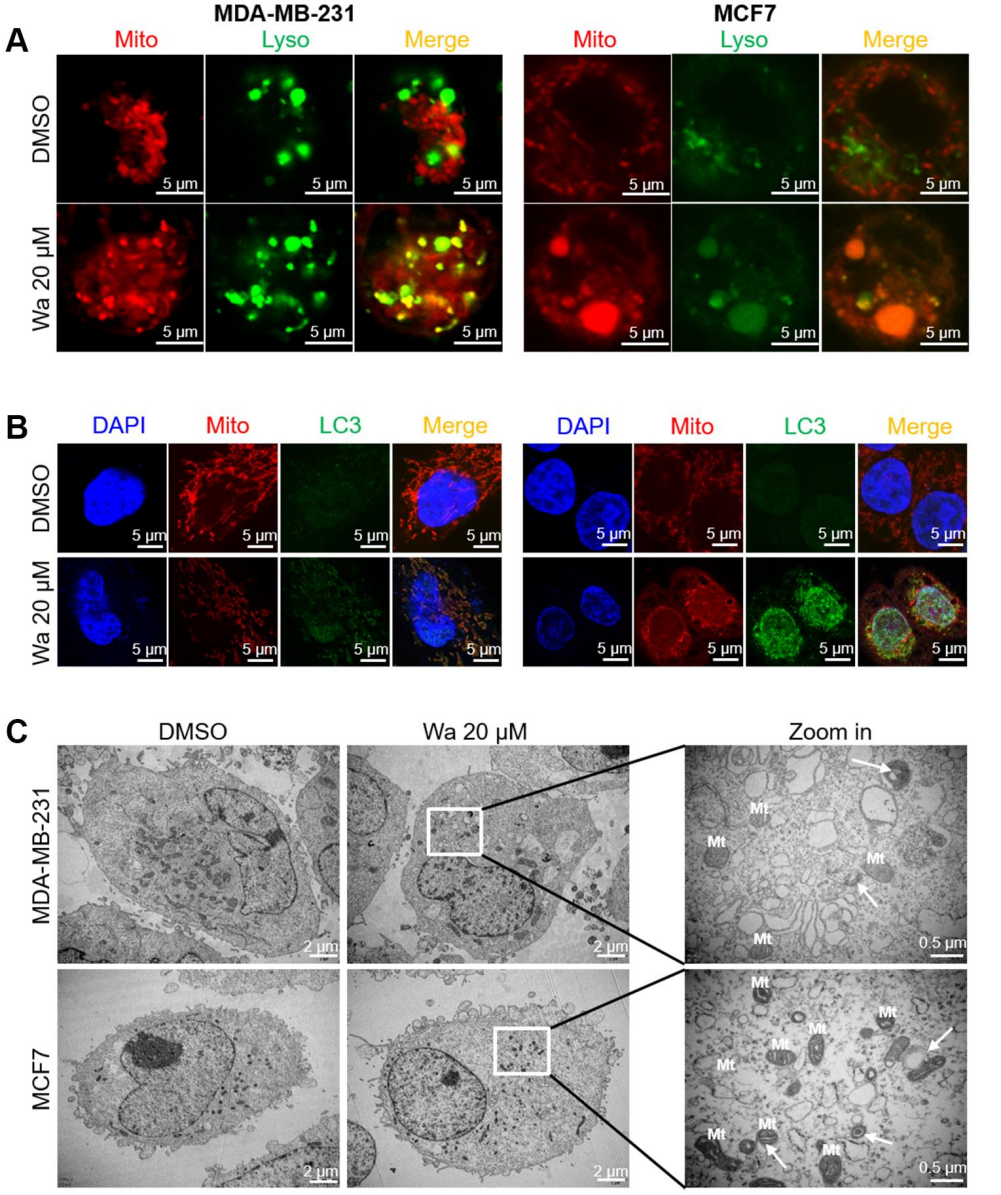
**Warangalone induces cell mitophagy.** MDA-MB-231 and MCF7 cells were treated with 20 μM warangalone for 12 h. A CLSM was used to observe co-staining. (**A**) Co-staining of lysosomes (LysoTracker™ Green DND) and mitochondria (MitoTracker™ Red CMXRos). (**B**) Co-staining of LC3 (labeled with Alexa Fluor 488) and mitochondria (MitoTracker™ Red CMXRos). (**C**) Transmission electron microscopy was used to observe mitophagy. Nuclei labeled with DAPI. Arrows indicate mitophagy.

Mitophagy, a form of autophagy, is initiated to specifically remove damaged mitochondria by lysosomes. As shown in Figures 2, 4, treatment with warangalone for 12 h resulted in mitochondrial damage and induced autophagy. Mitophagy was also studied with a CLSM and TEM. As shown in Figure 5A, treatment with warangalone for 12 h resulted in significant co-localization between mitochondria and lysosomes in MDA-MB-231 and MCF7 cells, indicating that warangalone induced mitophagy. Additionally, the number of lysosomes increased in the cells treated with warangalone, which indicated that warangalone induced autophagy. This finding is consistent with those shown in Figure 4. In addition, significant co-localization between LC3 and mitochondria was observed in MDA-MB-231 and MCF7 cells treated with warangalone (Figure 5B), which further proved that warangalone induced mitophagy.

Mitophagy is a dynamic process in which autophagosomes engulf damaged mitochondria. As shown in Figure 5C, after treatment with warangalone for 12 h double-layer vesicles (called “autophagosomes”) were observed around the damaged mitochondria in MDA-MB-231 and MCF7 cells, which might be autophagosomes engulfing damaged mitochondria.

### Mitophagy is activated through the PINK1/Parkin pathway

**Figure 6:**
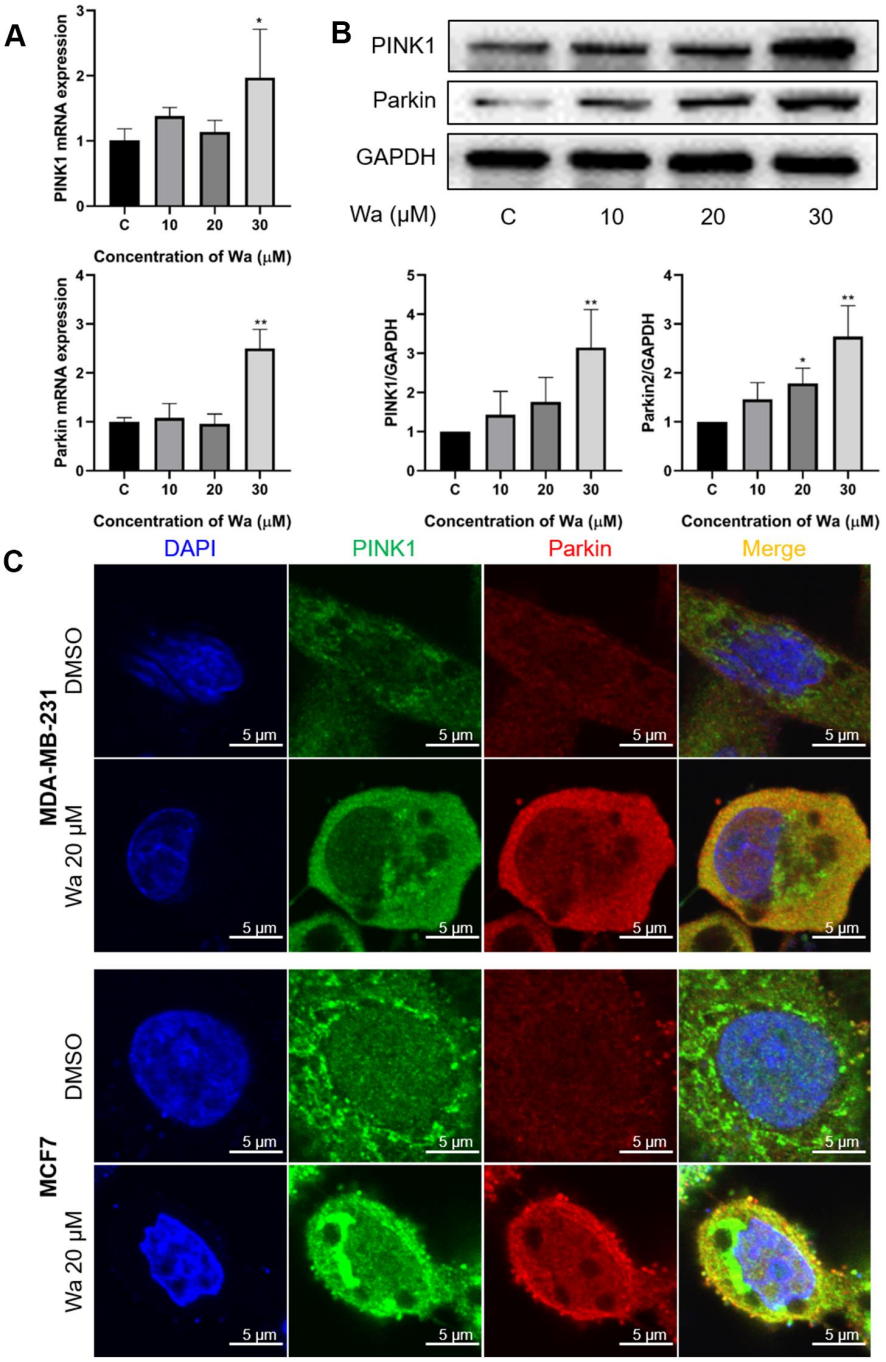
**Mitophagy is activated through the PINK1/Parkin pathway.** (**A**, **B**) MDA-MB-231 cells were treated with the indicated concentrations of warangalone for 12 h. The mRNA expressions of PINK1 and Parkin were measured by PCR (**A**), and protein expressions by Western blotting (**B**). GAPDH was used as the loading control. (**C**) MDA-MB-231 cells were treated with 20 μM warangalone for 12 h, and then immunostained with PINK1 and Parkin antibody and stained with DAPI. A CLSM was used to observe the fluorescence expression. Parkin labeled with Alexa Fluor 594 emits red, PINK1 labeled with Alexa Fluor 488 emits green, and nuclei labeled with DAPI emit blue. One-way ANOVA was used for statistical analysis (n ≥ 3). *P < 0.05, **P < 0.01, ***P < 0.001 compared to the respective control group.

Many studies have reported that mitophagy is activated through the PINK1/Parkin signaling pathway in cancer cells [23, 25]. Under stress, PINK1 is translocated and stabilized on the outer mitochondrial membrane (OMM) to recruit Parkin. Subsequently, PINK1 phosphorylates the poly-Ub chains, which are formed through ubiquitination of several components on the OMM by Parkin, thereby initiating mitophagy [22, 26]. Thus, PCR and Western blotting were used to determine if warangalone induced mitophagy via the PINK1/Parkin signaling pathway. As shown in Figure 6A, 30 μM warangalone significantly increased the mRNA expression levels of PINK1 and Parkin in MDA-MB-231 cells. Besides, warangalone increased the expression of PINK1 and Parkin in a dose-dependent manner (Figure 6B). Warangalone also increased the co-localization of PINK1 and Parkin in MDA-MB-231 and MCF7 cells (Figure 6C), indicating that mitophagy was activated through the PINK1/Parkin pathway.

### The inhibition of autophagy or mitophagy promotes apoptosis

**Figure 7:**
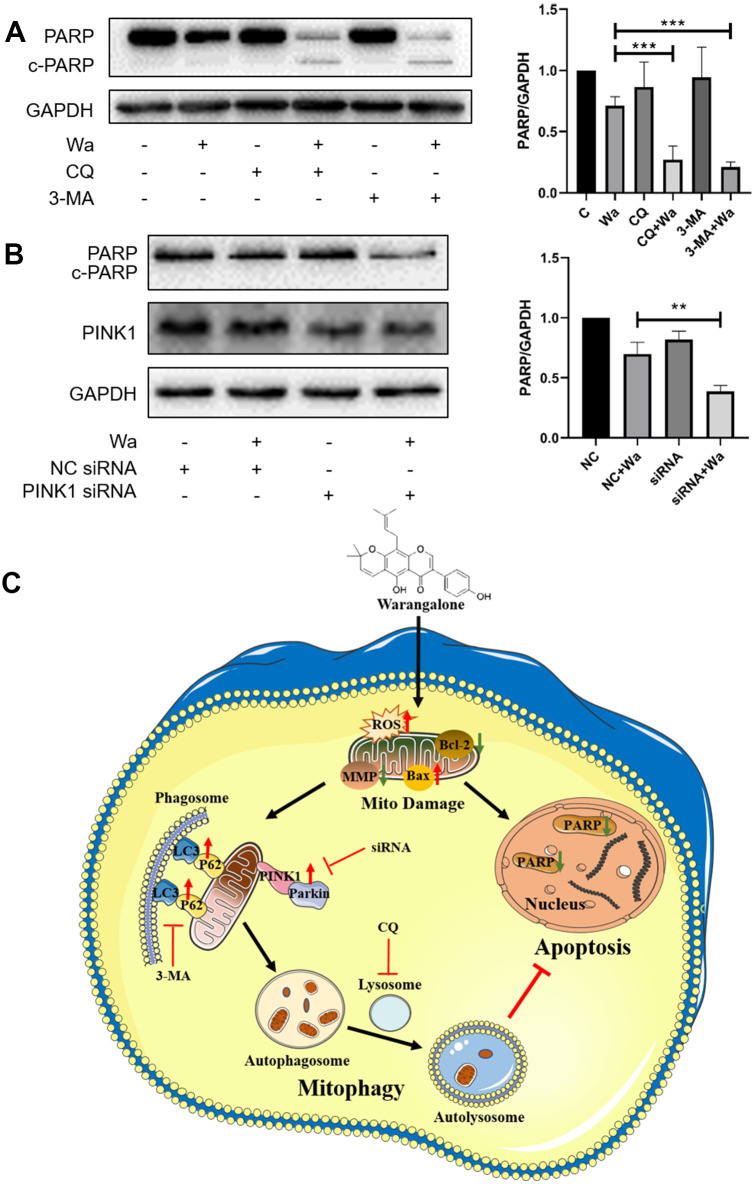
**Inhibition of autophagy or mitophagy promotes apoptosis.** (**A**) MDA-MB-231 cells were pretreated with CQ or 3-MA for 1 h, and the treated with 20 μM of warangalone for 12 h. Representative Western blots showed the expression of PARP. GAPDH was used as the loading control. Protein expression of PARP was quantified by densitometry and normalized to GAPDH (ratio PARP:GAPDH). (**B**) PINK1 was knockdown by PINK1 siRNA in MDA-MB-231 cells. After treatment with 20 μM warangalone for 12 h, Western blotting was used to detect the expression of PINK1 and PARP. GAPDH was used as the loading control. Protein expression of PARP was quantified by densitometry and normalized to GAPDH (ratio PARP:GAPDH). (**C**) Illustration of the possible mechanism by which warangalone acts on breast cancer cells.

To explore the relations between apoptosis and autophagy, CQ and 3-MA were used to inhibit autophagy, and Western blotting was performed to detect the expression of PARP. As shown in Figure 7A, the expression of PARP in MDA-MB-231 cells treated with CQ + warangalone or 3-MA + warangalone was decreased significantly compared to that in the warangalone alone treatment group, indicating that inhibition of autophagy promoted apoptosis. Apart from this, PINK1 siRNA was used to knockdown the expression of PINK1 in order to study whether the inhibition of mitophagy also promoted apoptosis. As shown in Figure 7B, compared to MDA-MB-231 cells treated with NC + warangalone the expression of PARP was significantly decreased in those treated with PINK1 siRNA + warangalone, indicating the inhibition of mitophagy also promoted apoptosis. In addition, the results of Annexin V-FITC/PI assay also confirmed that the inhibition of mitophagy promoted apoptosis by PINK1 siRNA. Compared to MDA-MB-231 cells treated with NC + warangalone the apoptosis rate increased significantly in those treated with PINK1 siRNA + warangalone (Supplementary Figure 1).

## DISCUSSION

Advances in the treatment of breast cancer, especially for ER^+^ and HER2^+^ disease, the 5-year relative survival rate of breast cancer patients has increased markedly. However, little progress has been made in the treatment of TNBC, and treatment is primarily limited to chemotherapy [27]. Previous studies have shown that warangalone has anti-cancer activity against leukemia [28] and melanoma [17]. Jiang et al. [18] reported that warangalone effectively inhibited the viability of MCF7 (Luminal A-ER^+^) cells in a dose-dependent manner. However, little study has been done with respect to the anti-cancer activity of warangalone in TNBC. In this study, we found that warangalone significantly inhibited cell viability of 3 breast cancer cell lines: MDA-MB-231 (TNBC), ZR75-1 (Luminal B), and SKBR3 (HER2^+^), but had no obvious toxicity to normal breast cells. In addition, we found that warangalone led to mitochondrial damage via ROS and induced PINK1/Parkin-mediated mitophagy and mitochondrial apoptosis. But autophagy/mitophagy protected against warangalone induced mitochondrial apoptosis. Taken together, these findings suggested that a combination of warangalone and autophagy/mitophagy inhibitors might be a promising treatment for TNBC.

Properly functioning mitochondria is crucial for maintaining cellular homeostasis, and thus targeting damage mitochondria in cancer cells has become an important area of anti-cancer drug development. Our results indicated that warangalone could damage mitochondria in breast cancer cells. We also found that the IC50 of warangalone was about 20 μM, which is similar to that of the conventional chemotherapeutic reagent 5-fluorouracil in breast cancer cells [25, 26]. Thus, the food-derived isoflavone (warangalone) may be a good chemotherapy candidate with fewer side effects than 5-fluorouracil.

The multi-ring structure of the flavonoids is supposed to be an anti-inflammation drug which inhibiting the generation of ROS in the inflammation. However, our results showed that warangalone promoted the generation of ROS in breast cancer cells. Even though in normal cells, ROS generate in the process of mitochondrial metabolism and homeostasis. And mitochondrial channels could play an important role in adaptive and maladaptive responses to redox stress [29]. Once mitochondrial channels were activated, mito-ROS would be released. However, longer mitochondrial channels openings may sustainedly release ROS, leading to destruction of mitochondria and further releasing ROS into cytoplasm [29]. And we found that mitochondrial damage caused by warangalone was due to ROS. Therefore, warangalone may activate mitochondrial channels to release a ROS burst resulting in mitochondrial damage, which is similar to the results reported in other studies [30].

Once mitochondrial damage occurs, cells initiate mitophagy to remove damaged mitochondria, and even induce mitochondrial apoptosis causing cell death [23]. Mitophagy, a form of autophagy, specifically removes damaged mitochondria by lysosomes. When mitophagy is activated, cells promote mitochondrial fission to divide mitochondria into fragments which assists autophagosomal packaging. Next, autophagosomes, in which LC3 binds with P62, fuse with lysosomes to form autolysosomes [22, 26]. Our results showed that PINK1/Parkin-mediated mitophagy was activated in breast cancer cells after treatment with warangalone for 12 h. However, mitophagy usually acts as a protective factor to maintain cell survival, and study has reported that PINK1/Parkin-medicated mitophagy led to drug resistance [23]. Therefore, we further studied if warangalone induced mitochondrial apoptosis.

Apoptosis, a process of cell death to eliminate damaged cells, plays a vital role in the development and progression of tumors. However, inhibition of apoptosis may lead to drug resistance, thereby limiting the anti-cancer activity of chemotherapy [31]. We found that when the treatment time with warangalone was increased to 24 h, mitochondrial apoptosis was induced via an increase of the BAX/BCL-2 ratio. Taken together, warangalone causes mitochondrial damage via production of ROS, thereby triggering PINK1/Parkin-mediated mitophagy and inducing mitochondrial apoptosis.

Many studies have shown that the inhibition of autophagy promotes apoptosis [32, 33]. Our results also showed that an autophagy inhibitor (CQ or 3-MA) promoted warangalone-induced apoptosis, which indicated that warangalone-induced autophagy protected cancer cells from death. Mitophagy, as a type of autophagy, selectively clears damaged mitochondria and maintains cell homeostasis. Some studies also indicated that mitophagy suppressed apoptosis and maintained cells. Mentioned above, our results showed that warangalone led mitochondrial damage and further activated PINK1/Parkin-mediated mitophagy. Besides, we further found that knock-down of PINK1 activated PARP to cleave, which suggested the inhibition of mitophagy also promoted apoptosis. In addition, the inhibition of autophagy or mitophagy has been shown to enhance the anti-cancer activity of drugs [34]. These prior studies support our results that a combination of warangalone and autophagy/mitophagy inhibitors may be a promising treatment for TNBC.

In summary (Figure 7C), warangalone effectively inhibits the viability of breast cancer cells. Warangalone causes mitochondrial damage via production of ROS, thereby triggering PINK1/Parkin-mediated mitophagy and inducing mitochondrial apoptosis. Moreover, autophagy or mitophagy protects against warangalone-induced mitochondrial apoptosis. Taken together, our results suggest that a combination of warangalone and autophagy/mitophagy inhibitors may be a promising treatment for TNBC.

## MATERIALS AND METHODS

### Reagents and chemicals

Warangalone was purchased from Shanghai Yuanye Bio-Technology (Shanghai, China); molecular formula, C_25_H_24_O_5_; molecular weight, 404.462; analysis of standard substance by high-performance liquid chromatography (HPLC) ≥ 98%. Dulbecco's modified Eagle’s medium (DMEM), fetal bovine serum (FBS), phosphate-buffered saline (PBS), penicillin-streptomycin, trypsin, and TRIzol were purchased from Gibco-Invitrogen (Carlsbad, CA, USA). 3-(4,5-dimethyl-2-thiazolyl)-2,5-diphenyl-2-H-tetrazolium bromide (MTT), 4',6-diamidino-2-phenylindole (DAPI), monodansylcadaverine (MDC), dimethyl sulfoxide (DMSO), 3-methyladenine (3-MA) and chloroquine (CQ) were purchased from Sigma-Aldrich (St. Louis, MO, USA). Primary antibodies against GAPDH, LC3, BAX, BCL-2 and PARP were obtained from Cell Signaling Technology (Beverly, MA, USA). Antibodies against VDAC1 and PINK1 were purchased from ABclonal Technology (Boston, MA, USA). Antibodies against P62 and Parkin were purchased from Proteintech (Chicago, IL, USA). Hoechst 33342 solution was obtained from Beyotime Biotechnology (Shanghai, China). Cell cycle detection, reactive oxygen species (ROS) analysis, Annexin V-FITC/PI, and mitochondrial membrane potential detection kits were purchased from KeyGEN BioTECH (Nanjing, Jiangsu, China). MitoTracker Red CMXRos and LysoTracker™ Green DND-26 were obtained from Thermo Fisher Scientific (Waltham, MA, USA).

### Cell culture

The human invasive breast cancer cell line MDA-MB-231 (TNBC) and human noninvasive breast cancer cell lines MCF7 (Luminal A), ZR75-1 (Luminal B) and SKBR3 (HER2^+^) were obtained from American Type Culture Collection (Rockefeller, MD, USA). The cells were cultured in L15, MEM, RPMI-1640 or DMEM basic medium containing 10% FBS and 100 units/mL penicillin and 100 mg/mL streptomycin at 37° C in a 5% CO_2_ atmosphere.

### MTT assay

Cells were seeded in 96-well plates at a density of 1×10^4^ cells/well overnight. After treatment with various warangalone concentrations for 12, 24 and 48 h, the cells were incubated with 0.5 mg/mL MTT solution for 4 h. A microplate reader (ELX808, Biotek, UK) was used to detect the absorbance (optical density [OD]) at 490 nm after dissolving the formazan with DMSO. Cell viability was calculated using the following formula:

Cell viability (%) = [(A490_sample_ - A490_blank_) / (A490_control_ - A490_blank_)] × 100.

### Cell morphology analysis

MDA-MB-231 and MCF7 cells were seeded in 6-well plates at 1×10^6^ cells/mL and incubated overnight. The cells were then treated with 0, 10, 20 and 30 μM warangalone for 24 h. After the cells were washed with PBS twice, a fluorescence microscope (IX73, Olympus, Japan) was used to observe the cell morphology.

### Hoechst 33342 staining assay

MDA-MB-231 and MCF7 cells were seeded in 6-well plates at 1×10^6^ cells/mL and cultured overnight. After treatment with warangalone for 24 h, the cells were incubated with Hoechst 33342 solution (5 μg/mL) for 15 min. A fluorescence microscope (IX73) was used to observe the fluorescence level at an excitation wavelength of 346 nm, and an emission wavelength of 460 nm.

### Annexin V-FITC/PI assay

Cells were treated with warangalone for 24 h, and then harvested and washed for subsequent experiments according to the kit manufacturer’s instructions. After the cells were suspended in binding buffer, Annexin V-FITC and PI solutions were added, and the cells were incubated for 30 min. Flow cytometry (BD LSRFortessa X-20, USA) was used to detect the apoptosis rate, and the results were analyzed using FlowJo software, version 10.

### Western blotting

Total protein was extracted using RIPA lysis buffer containing protease inhibitor and phosphatase inhibitor (BestBio, Shanghai, China). Protein was loaded onto SDS-PAGE gels, separated through electrophoresis, and transferred to PVDF membranes (Merck KGaA, Darmstadt, Germany). The membranes were incubated with the target primary antibody (PARP, BAX, BCL-2, LC3, P62, PINK1, Parkin and GAPDH) at 4° C overnight and then with the secondary antibody (1:5000, Santa Cruz Biotechnology, TX, USA) at room temperature for 2 h. Immunoblots were visualized using enhanced chemiluminescence (Fdbio Science, Hangzhou, China) and a Tanon-S200 imaging system (Shanghai, China). Bands were quantified and normalized to the loading control.

### MDC staining assay

MDA-MB-231 and MCF7 cells were seeded on 6-well plates at 1×10^6^ cells/mL and cultured overnight. After treatment with warangalone for 12 h, the cells were incubated with 0.05 mM MDC solution for 30 min. A fluorescence microscope (IX73, Olympus, Japan) was used to observe the fluorescence level at an excitation wavelength of 335 nm and an emission wavelength of 518 nm.

### Fluorescence staining, immunofluorescence and laser confocal imaging

To detect mitochondrial morphology and the co-localization of mitochondria and lysosomes, cells were seeded in laser confocal dishes overnight and treated with warangalone for 12 h. MitoTracker™ Red CMXRos (excitation/emission wavelength: 579 nm/599 nm) and LysoTracker™ Green DND-26 (excitation/emission wavelength: 504 nm/511 nm) were used to stain the mitochondria for 30 min and lysosomes for 1 h, respectively. A confocal laser scanning microscope (CLSM) (LSM 880 with Airyscan, Zeiss, Germany) was used to observe the mitochondria and lysosomes.

To further study the expression of LC3 and the co-localization of LC3 and mitochondria, the cells were incubated with or without MitoTracker™ Red CMXRos (excitation/emission wavelength: 579 nm/599 nm) for 30 min after treatment with warangalone for 12 h. The cells were fixed with 4% paraformaldehyde for 15 min at room temperature, treated with precooled methanol for 15 min at -20° C, and incubated with primary antibody against LC3 overnight at 4° C. Then, the cells were incubated with goat anti-rabbit IgG-Alexa Fluor 488 (excitation/emission wavelength: 496 nm/519 nm) for 1 h and stained with DAPI (excitation/emission wavelength: 359 nm/461 nm) for 15 min. A CLSM was used to observe and photograph the cells.

In addition, to measure the expression and co-localization of PINK1 and Parkin, after treatment with warangalone for 12 h, the cells were fixed with paraformaldehyde, treated with methanol, and incubated with primary antibodies against PINK1 and Parkin. Then, the cells were incubated with goat anti-rabbit IgG-Alexa Fluor 488 and goat anti-rabbit IgG-Alexa Fluor 594, and subsequently stained with DAPI solution. A CLSM was used to observe and photograph the cells.

### Transmission electron microscopy (TEM)

After treatment with warangalone for 12 h, cells were harvested with a cell scraper and fixed with 2.5% ice-cold glutaraldehyde for 30 min. The cells were post-fixed in osmium tetroxide and embedded in Spurr’s Epon. A transmission electron microscope (7500, Hitachi, Japan) was used to observe the cells.

### Mitochondrial membrane potential (MMP)

Cells were seeded in 6-well plates overnight and treated with warangalone for 12 h. Then, the cells were incubated with JC-1 solution for 20 min according to the manufacturer’s instructions. After the cells were washed with incubation buffer, a fluorescence microscope (IX73, Olympus, Japan) was used to observe the level of fluorescence.

Besides, after treatment with warangalone cells were harvested with trypsin, washed with PBS, and incubated with JC-1 solution for 30 min. Flow cytometry (BD LSRFortessa X-20, USA) was then performed to detect the JC-1 signal.

### ROS measurement

Cells were seeded in 6-well plates overnight and treated with warangalone for 12 h. After the cells were incubated with 10 μM dichlorodihydrofluorescein diacetate (DCFH-DA) for 30 min, a fluorescence microscope (IX73) was used to monitor ROS generation. Additionally, the cells were seeded in black 96-well plates at a density of 1×10^4^ cells/well overnight and treated with warangalone for 12 h. After the cells were incubated with 10 μM DCFH-DA for 30 min, a fluorescence spectrophotometer (A5082, Tecan Spark, Australia) was used to monitor ROS generation.

### Real-time PCR

Total RNA was isolated and purified using TRIzol, phenol, chloroform and ethanol. After RNA reverse-transcription, the cDNA was mixed with primers and SYBR Real-time PCR buffer (Takara, Tokyo). Then, a real-time PCR detection system (CFX Connect™, BIORAD, USA) was used to detect gene amplification. The primer sequences of GAPDH, Parkin, and PINK1 were:

GAPDH forward: CAAATTCCATGGCACCGTCA,

GAPDH reverse: ATCTCGCTCCTGGAAGATGG;

PINK1 forward: GGACACGAGACGCTTGCA,

PINK1 reverse: TTACCAATGGACTGCCCTATCA;

Parkin forward: GTGTTTGTCAGGTTCAACTCCA,

Parkin reverse: GAAAATCACACGCAACTGGTC.

### RNAi

According to the manufacturer’s protocol, the PINK1 siRNA sequence at a final concentration of 50 nM was transfected into the cells with riboFECT CP transfection reagent. After transfection for 48 h, cells were treated with 20 μM warangalone for 12 h. Western blotting was used to detect the expression level of PINK1, PARP and GAPDH.

### Statistical analysis

Data were assessed using one-way analysis of variance (ANOVA) in conjunction with Duncan’s new multiple-range test. Statistical analysis was performed using SPSS version 25.0 software. Values of P < 0.05 were considered to indicate a statistically significant difference.

## Supplementary Material

Supplementary Figure 1
