# Supplementary material for: PINK1/Parkin-mediated mitophagy inhibits warangalone-induced mitochondrial apoptosis in breast cancer cells
Source: Aging (Albany NY). 2021 Apr 30;13(9):12955–72. doi: 10.18632/aging.202965 (PMC8148507; doi:10.18632/aging.202965)
Supplement: Supplementary Figure 1 [file aging-13-202965-s001.pdf]

SUPPLEMENTARY FIGURE

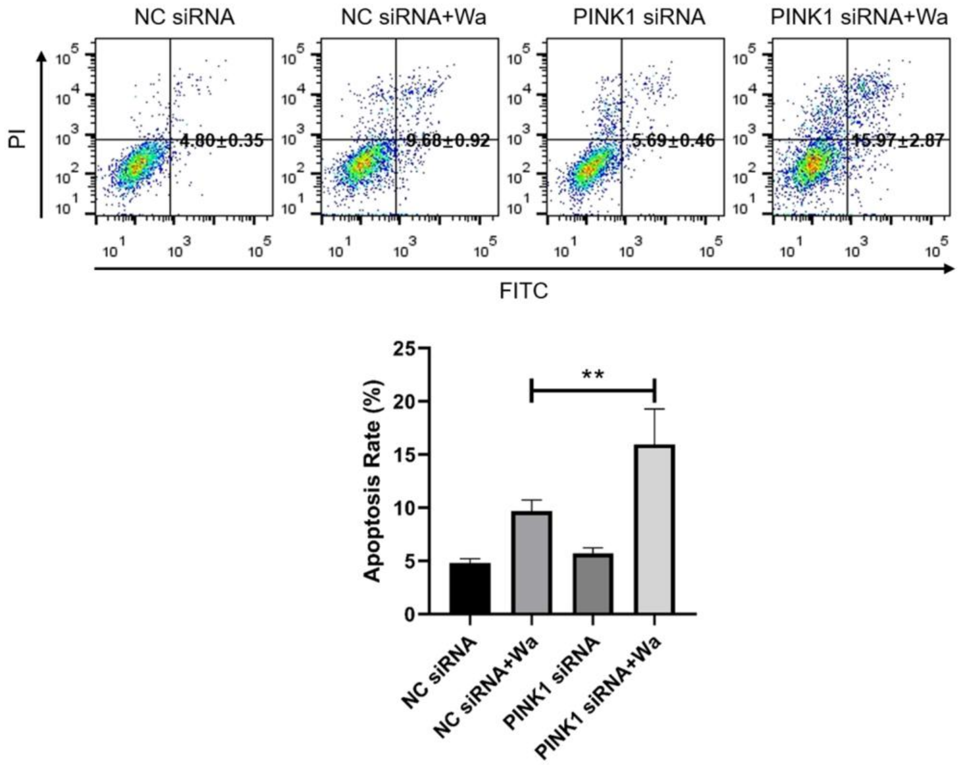

Supplementary Figure 1. Inhibition of mitophagy promotes apoptosis. PINK1 was knockdown by PINK1 siRNA in MDA-MB-231 cells. After treatment with 20 μM warangalone for 12 h, Annxin V-FITC/PI assay was used to detect the apoptosis rate. One-way ANOVA was used for statistical analysis (n ≥ 3). *P < 0.05, **P < 0.01, ***P < 0.001 compared to the respective control group.
